# Supplementary material for: Difference in Methylation and Expression of Brain-Derived Neurotrophic Factor in Alzheimer’s Disease and Mild Cognitive Impairment
Source: Biomedicines. 2023 Jan 17;11(2):235. doi: 10.3390/biomedicines11020235 (PMC9953261; doi:10.3390/biomedicines11020235)
Supplement: Supplementary file 1 [file biomedicines-11-00235-s001.zip › biomedicines-2100243-supplementary.pdf]

biomedicines

MDPI

Table S1. Amplicon primer sequences and annealing temperatures for *BDNF* and *COMT* genes. In grey shade are Illumina universal sequences that are followed by the sequence-specific primers aligning on the target bisulfite converted DNA sequence.

**Amplicon**	**Forward or reverse primer**	**Primer Sequence**	**Annealing temperature / °C**
COMT_1	Forward primer	**TCGTCGGCAGCGTCAGATGTGTATAAGAGA-CAG**GTTTTTAATTTTGTATAGGTAAGAT	54
	Reverse primer	**GTCTCGTGGGCTCGGAGATGTGTATAAGAGACAG-**TACCCTCCCTACCCACAAC	
COMT_2	Forward primer	**TCGTCGGCAGCGTCAGATGTGTATAAGAGA-CAG**GGTTATTTGTGGTTAGAAGTAGTT	54
	Reverse primer	**GTCTCGTGGGCTCGGAGATGTGTATAAGAGA-CAG**ACTACCCCAAAAACCCCAC	
COMT_4	Forward primer	**TCGTCGGCAGCGTCAGATGTGTATAAGAGA-CAGNN**TGTAGGAGGAGTATAGAGTATTGG	62
	Reverse primer	**GTCTCGTGGGCTCGGAGATGTGTATAAGAGA-CAGNN**TCATAACCCACTCCTTCTACT	
BDNF_1	Forward primer	**TCGTCGGCAGCGTCAGATGTGTATAAGAGACAG-**TGAGGGTAGGTAAAGGGTAGT	55.3
	Reverse primer	**GTCTCGTGGGCTCGGAGATGTGTATAAGAGA-CA**ACTCTCCCAAAAAACCCTAC	
BDNF_2	Forward primer	**TCGTCGGCAGCGTCAGATGTGTATAAGAGACAG-**TTATATAGGTTTTTGTGGGTAATTAG	55
	Reverse primer	**GTCTCGTGGGCTCGGAGATGTGTATAAGAGA-CAG**AAAAAAAAAAAAACTTCTTAAAAAAAT	
BDNF_3	Forward primer	**TCGTCGGCAGCGTCAGATGTGTATAAGAGACAG-**TTTTTAGTTATGATGGGGGAGG	58
	Reverse primer	**GTCTCGTGGGCTCGGAGATGTGTATAAGAGACAG-**CAAATCACACCTAAAACTCC	
BDNF_4	Forward primer	**TCGTCGGCAGCGTCAGATGTGTATAAGAGACAG-**GAGTTTATTTAGTATTTTGGATAGA	55.3
	Reverse primer	**GTCTCGTGGGCTCGGAGATGTGTATAAGAGA-CAG**AAAAATCTATTCCAACCTACACC	
BDNF_5	Forward primer	**TCGTCGGCAGCGTCAGATGTGTATAAGAGACAG-TTTTTTTAAGGGAAGGGGAGTT**	54
	Reverse primer	**GTCTCGTGGGCTCGGAGATGTGTATAAGAGA-CAGAAACTAAAAATATTCTTCTCCACC**	
BDNF_6	Forward primer	**TCGTCGGCAGCGTCAGATGTGTATAAGAGACAGA-TAGAGTTATTAATTAGTTGGA**	55
	Reverse primer	**GTCTCGTGGGCTCGGAGATGTGTATAAGAGACAG-TAAATCCCTAAACTCCCTAAAA**	
BDNF_7	Forward primer	**TCGTCGGCAGCGTCAGATGTGTATAAGAGA-CAGGGTTTTAATGAGATATTTAT**	58
	Reverse primer	**GTCTCGTGGGCTCGGAGATGTGTATAAGAGA-CAGAAAATCCCCCAATCAACTCTCT**	
BDNF_8	Forward primer	**TCGTCGGCAGCGTCAGATGTGTATAAGAGA-CAG**AAATGTTGTTATTATTTTGATTGAATT	54
	Reverse primer	**GTCTCGTGGGCTCGGAGATGTGTATAAGAGA-CAG**AACACCCAAATTCTCTAAAAAA	
BDNF_9	Forward primer	**TCGTCGGCAGCGTCAGATGTGTATAAGAGA-CAGNN**TTTTTTAGAGAATTTGGGTGT	56.7
	Reverse primer	**GTCTCGTGGGCTCGGAGATGTGTATAAGAGA-CAGNN**AAACCTATCCTCACCTCCT	

Table S2. Correlation of *BDNF* expression with demographic and clinical characteristics of subjects divided by diagnosis into subjects with MCI and subjects with AD.

Characteristics	MCI		AD	
	r_s	p	r_s	p
Age (years)	-0.014	0.897	0.089	0.450
BMI (kg/m^2)	0.028	0.798	-0.071	0.548
Waist circumference (cm)	-0.009	0.935	-0.134	0.256
Total cholesterol (mmol/l)	0.079	0.470	-0.063	0.592
HDL-cholesterol (mmol/l)	0.255	0.018	-0.168	0.152
LDL-cholesterol (mmol/l)	-0.023	0.834	-0.056	0.634
Triglycerides	-0.010	0.930	0.099	0.401
Blood glucose (mmol/l)	0.036	0.742	0.121	0.306

AD, Alzheimer's Disease; HDL, High-Density Lipoproteins; BMI, Body Mass Index; LDL, Low-Density Lipoproteins; MCI, Mild Cognitive Impairment; r_s, Spearman's Rank Correlation Coefficient

Table S3. Correlation of *COMT* expression with demographic and clinical characteristics of subjects divided by diagnosis into subjects with MCI and subjects with AD.

Characteristics	MCI		AD	
	r_s	p	r_s	p
Age (years)	0.073	0.507	-0.151	0.057
BMI (kg/m^2)	-0.012	0.915	0.184	0.117
Waist circumference (cm)	-0.082	0.451	0.176	0.134
Total cholesterol (mmol/l)	-0.096	0.379	0.036	0.760
HDL-cholesterol (mmol/l)	-0.031	0.775	-0.118	0.317
LDL-cholesterol (mmol/l)	-0.013	0.905	0.062	0.597
Triglycerides	-0.205	0.058	0.102	0.386
Blood glucose (mmol/l)	-0.138	0.204	-0.058	0.626

AD, Alzheimer's Disease; HDL, High-Density Lipoproteins; BMI, Body Mass Index; LDL, Low-Density Lipoproteins; MCI, Mild Cognitive Impairment; r_s, Spearman's Rank Correlation Coefficient
